# Synergistic impact of *Serendipita indica* and *Zhihengliuella* sp. ISTPL4 on the mitigation of arsenic stress in rice

**DOI:** 10.3389/fmicb.2024.1374303

**Published:** 2024-05-29

**Authors:** Neha Sharma, Gaurav Yadav, Jaagriti Tyagi, Ajay Kumar, Monika Koul, Naveen Chandra Joshi, Abeer Hashem, Elsayed Fathi Abd_Allah, Arti Mishra

**Affiliations:** ^1^Amity Institute of Microbial Technology, Amity University, Noida, Uttar Pradesh, India; ^2^Amity Institute of Biotechnology, Amity University, Noida, Uttar Pradesh, India; ^3^Department of Botany, Hansraj College, University of Delhi, New Delhi, India; ^4^Botany and Microbiology Department, College of Science, King Saud University, Riyadh, Saudi Arabia; ^5^Plant Production Department, College of Food and Agricultural Sciences, King Saud University, Riyadh, Saudi Arabia

**Keywords:** Arsenic Stress, phytohormones, plant growth, Serendipita indica, stress enzymes, Zhihengliuella sp. ISTPL4

## Abstract

Arsenic (As) is a highly toxic metal that interferes with plant growth and disrupts various biochemical and molecular processes in plants. In this study, the harmful effects of As on rice were mitigated using combined inoculation of a root endophyte *Serendipita indica* and an actinobacterium *Zhihengliuella* sp. ISTPL4. A randomized experiment was conducted, in which rice plants were grown under controlled conditions and As-stressed conditions. The control and treatment groups consisted of untreated and non-stressed plants (C1), treated and non-stressed plants (C2), stressed and untreated plants (T1), and stressed and treated plants (T2). Various phenotypic characteristics such as shoot length (SL), root length (RL), shoot fresh weight (SFW), root fresh weight (RFW), shoot dry weight (SDW), and root dry weight (RDW) and biochemical parameters such as chlorophyll content, protein content, and antioxidant enzymatic activities were evaluated. The activity of various antioxidant enzymes was increased in T2 followed by T1 plants. Furthermore, high concentrations of phytohormones such as ethylene (ET), gibberellic acid (GA), and cytokinin (CK) were found at 4.11 μmol mg^−1^, 2.53 μmol mg^−1^, and 3.62 μmol mg^−1^ of FW of plant, respectively. The results of AAS indicated an increased As accumulation in roots of T2 plants (131.5 mg kg^−1^) than in roots of T1 plants (120 mg kg^−1^). It showed that there was an increased As accumulation and sequestration in roots of microbial-treated plants (T2) than in uninoculated plants (T1). Our data suggest that this microbial combination can be used to reduce the toxic effects of As in plants by increasing the activity of antioxidant enzymes such as SOD, CAT, PAL, PPO and POD. Furthermore, rice plants can withstand As stress owing to the active synthesis of phytohormones in the presence of microbial combinations.

## 1 Introduction

The discharge of substances such as arsenic (As), chromium (Cr), and cadmium (Cd) into the environment from various sources such as agricultural chemicals, industrial activities, and wastewater has notable implications. These toxic metals can adversely affect the microbial composition of the soil, leading to a chain of effects on soil properties and agricultural productivity (Alengebawy et al., 2021). As is a toxic metalloid that can be released from both natural and human-made sources. When groundwater is utilized for irrigation, As accumulates and intensifies in crops along the food chain. This elevation in surface levels of the As poses risks to human health, potentially leading to cognitive impairments and an increased risk of cancer (Genchi et al., 2022). Additionally, As hampers photosynthesis and diminishes transpiration efficiency in plants. Its accumulation in plants is influenced by their genetic makeup, and since it also builds up in soil, remediation efforts become challenging, rendering a significant portion of agricultural land that is unsuitable for cultivation (Gupta et al., 2022).

Plants typically absorb As in both pentavalent As (V) and trivalent As (III) forms. Among them, As(III) is more toxic and can interrupt various cellular processes and has been shown to react with sulfhydryl groups of proteins and enzymes, thereby leading to inhibition of cellular processes and eventually resulting in impaired plant development (Shri et al., 2009). To reduce As stress, plants have evolved several molecular processes, such as synthesis of phytochelatins (PC), glutathione, and antioxidant enzyme systems (Das and Roychoudhury, 2014). Since rice is a primary food source for world’s population, it is crucial to reduce As levels in rice (Sharma et al., 2024). One potential sustainable strategy we can employ to address the issue is utilizing microorganisms, either separately or in combination. Several studies suggest that microbial consortia tend to outperform individual strains in various tasks. Examples include breaking down plant polymers such as cellulose, executing multi-step processes, metabolizing complex substances, and ensuring stability in volatile environments. The utilization of microbial consortia is necessary because certain activities cannot be accomplished by a single microbe alone (Kuiper et al., 2004; Santoyo et al., 2021). These microorganisms also diminish the harmful effects of heavy metals by transforming them into less harmful forms and storing them in vacuoles, which enhance metal sequestration (Tarekegn et al., 2020; Skuza et al., 2022; Jeyakumar et al., 2023).

Plants and microorganisms could survive in an environment polluted with metals by releasing growth-promoting substances, including phytohormones that act as signaling molecules and aid in plant growth (Tiwari et al., 2020). Microorganisms colonize rhizosphere and help in abiotic stress tolerance by secreting various compounds, such as indole acetic acid (IAA), antioxidants, volatile compounds, osmolytes, and exopolysaccharides (Rajkumar et al., 2009). Phytohormones involving IAA, gibberellin (GA), and brassinosteroid (BR) boost growth of plants in various biotic and abiotic stresses (Hirayama, 2022). These phytohormones act as signaling molecules in cascade of biological and enzymatic reactions (Saad et al., 2022; Yadav et al., 2023). These pathways regulate gene expression involved in plant defense by producing metallothioneins (MTs), phytochelatins (PCs), etc. Salicylic acid (SA) is chief phytohormone that regulates photosynthesis, osmoregulation, and antioxidant defense systems by switching on various enzymes involved in tolerance of abiotic stress (Kaya et al., 2019; Awad et al., 2022). Understanding the entirety of microbial communities and evaluating the capacity of each microorganism to break down soil contaminants are vital aspects for successful bioremediation, as highlighted by Jacques et al. (2008). It is essential to grasp how bacterial and fungal groups react to metal toxicity, their interplay, and involvement in reducing metal impacts, as emphasized by Yadav et al. (2023).

*S. indica* is a root endophytic fungus which is well known for its plant growth promotion ability, while *Zhihengliuella* sp. ISTPL4 is an actinobacterium isolated from Pangong lake, Ladakh (Varma et al., 2014; Mishra et al., 2020). It was observed that *S. indica* grew under several heavy metals, such as As and Cd (Mohd et al., 2017; Abedi et al., 2023). Similarly, growth of *Zhihengliuella* sp. ISTPL4 was also observed in the presence of Cr (Mishra et al., 2020). However, there are no previous experimental investigations utilizing this specific microbial combination for mitigating As in rhizospheric soil. Nevertheless, there are documented instances where the joint inoculation of *S. indica* and *Z.* sp. ISTPL4 has been reported to promote plant growth in rice (Sharma et al., 2023). In this study, we tested a combination of a fungus and a bacterium to examine their impact on the growth and development of rice plants in the context of As stress. The fungal and bacterial strains used in this study are *Serendipita indica* and *Zhihengliuella* sp. ISTPL4. We conducted a series of experiments both in the laboratory and pots, with the following specific objectives: **(i)** to evaluate the tolerance of *S. indica* and *Z*. sp. ISTPL4 to As *in vitro*, **(ii)** to examine the effect of this combination on rice growth under As stress, **(iii)** to measure the levels of various antioxidant enzymes (CAT, PAL, LOX, PPO, and SOD) and phytohormones in rice plants under both control and stress conditions (with and without microbial treatment), and **(iv)** to assess the impact of this combination on As accumulation in rice plants. Our results indicate that this combination of microbes has the potential to act as a bioinoculant, promoting plant growth under various abiotic stress conditions, thereby supporting sustainable agricultural practices.

## 2 Methodology

### 2.1 Microorganisms

*S. indica* was grown in Hill and Kaefer (H & K) medium by inoculating a 4-mm fungal disc of *S. indica* followed by keeping the culture at 28 ± 2° C for 15 days. Similarly, *Zhihengliuella* sp. ISTPL4 was also grown in Luria–Bertani broth by inoculating a single colony of *Z.* sp. ISTPL4 and keeping the bacterial culture in incubation at 28 ± 2° C for 24 h at 120 rpm.

The biocompatibility of *S. indica* and *Z.* sp. ISTPL4 was checked according to the methodology by Sharma et al. (2023). The interaction between both the microbes was analyzed on modified medium (a combination of Hill and Kaefer medium and Luria–Bertani agar in 1:3 ratio). A 4-mm fungal disc was inoculated followed by streaking a secondary culture of *Z.* sp. ISTPL4 on 5th day of fungal inoculation (dafi) around the periphery, and then, plates were kept for incubation at 30° C for 20 days (Hill and Kafer, 2001).

The heavy metal tolerance capability of the combined culture of *S. indica* and *Z.* sp. ISTPL4 was checked at varying range of As (0.2, 0.9, 1.4, 1.9, 2.4, and 2.6 mM), to examine the effect of As on their growth. Medium used for their growth was Hill and Kaefer agar in combination with Luria–Bertani agar (1:3)(Hill and Kafer, 2001; Sharma et al., 2023, 2024).

### 2.2 *In vitro* rice experiment

An *in vitro* experiment was performed to check the growth of seedlings of *Oryza sativa* (Taipei) under varying concentrations of As. Germination of rice seedlings was checked in half-strength Murashige and Skoog (MS) medium followed by adjusting pH of medium at 5.8. The concentration range of As was from 0.2 to 1.2 mM (Murashige, 1962).

### 2.3 Plant experimental conditions

Bioremediation capability of *S. indica* and *Z.* sp. ISTPL4 was determined by conducting a pot experiment in bio hardening laboratory (Amity Institute of Microbial Technology (AIMT), Amity University, Uttar Pradesh, India). *Oryza sativa* (Japonica rice, Taipei 309) seeds were collected from NIPGR, New Delhi, India. Seed sterilization was performed by adopting the methodology by Yadav et al. (2023). After seed sterilization, seeds were kept for germination on sterile sheet in Petri plate before sowing (for 3 days, 22°C in dark). Pre-germinated seeds were then added to a germination dish containing autoclaved soil. Heavy metal was mixed in autoclaved soil. The methodology for mixing heavy metal was adopted by Dabral et al. (2019). Sodium arsenate (200 mg kg^−1^ and 400 mg kg^−1^) was added to soil (mM unit was converted to mg kg^−1^). The process was conducted in plastic trays followed by keeping soil for incubation at 22°C for 60 days for immobilization and stabilization of metal. After that, soil was added to pots (height: 11 cm, Width: 9 cm, and soil capacity: 265gm). Pre-germinated seeds were then transferred to the soil. Four seeds were added to each pot. There were four sets of treatment (1) C1 (untreated and non-stressed plants), (2) C2 (treated and non-stressed plants), (3) T1 (stressed and untreated plants), and (4) T2 (stressed and treated plants), respectively.

### 2.4 Microbial inoculation

After 7–10 days of seed germination, 1 mL of spore of fungus with spore count of 4.85 × 10^7^ mL^−1^ was inoculated around the rhizospheric soil of respective pots. In total, 1 mL of bacterial culture (optical density 0.4) was inoculated in the respective plants at 5 dafi (Sharma et al., 2023).

### 2.5 Phenotypic traits

SL, RL, SFW, RFW, SDW, and RDW were checked for each plant (40 days of experiment). SDW and RDW were checked by keeping roots and shoots in hot air oven (48 h, 80° C) (Tyagi et al., 2023).

### 2.6 Root colonization

Root colonization was checked after 10 days of microbial inoculation by cleaning plant roots and cutting into small pieces. Excised root then heated in 10% KOH (potassium hydroxide) for 15 min. Roots were treated with 1 N HCl and then kept in a water bath (3 min, 40° C) and stained with trypan blue (0.02%) overnight. Excess stain was removed with the help of 50% lactophenol. Fungal spores were visualized at 10x magnification using Nikon light microscope (Nikon eclipse E600, Japan) (Dabral et al., 2020). Root colonization was checked using following formula:

Root colonization percentage = (No of roots fragments colonized/Total root fragments) x 100.

### 2.7 Biochemical traits

#### 2.7.1 Chlorophyll content

It was measured by adding 1 gm plant sample (leaf) with 10 mL acetone (80%) followed by incubation of 24 h in dark. Chl a and Chl b were calculated by taking optical density (OD) at 663 and 645 nm, respectively (Arora et al., 2016).


$$\text{Chl}\;a=\left(12.72_{A663}-2.59_{A645}\right)\times V/\left(m\times 1,000\right).$$

$$\text{Chl}\;b=\left(22.88_{A663}-4.67_{A645}\right)\times V/\left(m\times 1,000\right).$$

TotalChl(mg/g)=Chla+Chlb.

#### 2.7.2 Protein content

It was estimated using the methodology by Bradford (1976). This was performed by mixing 0.1 gm of dried plant sample in 0.01 M buffer (potassium phosphate). The resulting solution was then centrifuged (12,000 rpm, 4°C, 10 min). In total, 1 mL of Bradford reagent was mixed in 0.05 mL extracted sample followed by incubation at RT for 10 min. OD was checked at 595 nm (Shimadzu UV-1700, Tokyo, Japan) (Bradford, 1976).

#### 2.7.3 Total soluble sugar (TSS)

It was determined by adding 100 μL of plant methanolic extract to 3 mL of anthrone reagent, (200 mg anthrone, 100 mL of 72% H_2_SO_4_), followed by keeping it at incubation (10 min, 100° C) in water bath. OD of sample was measured at 620 nm. Standard glucose curve was plotted using glucose range (20– 400 μg mL ^-1^) to determine total soluble sugar (Wang et al., 2006).

#### 2.7.4 Phenolic and flavonoid content

The phenolic content was estimated by taking 100 mg of lyophilized sample of plant in 10 mL methanol to form even suspension, keeping it overnight in a shaker and sonicated (30 min). In total, 1.15 mL deionized water was mixed in 20 μL methanolic extract and 300 μL Folin–Ciocalteu (F-C) and 200 mM disodium carbonate and kept it for incubation at 40° C. OD of the resulting mixture was checked at 765 nm. Gallic acid standard curve was plotted (Gao et al., 2000).

Total flavonoid content was determined by adapting the methodology by Chang et al. (2002). It was performed by adding 500 μL of methanolic extract to 2% AlCl_3_ (aluminium chloride) followed by keeping it in incubation for 1 h. OD was measured at 420 nm (Chang et al., 2002).

#### 2.7.5 Malondialdehyde assay (MDA)

It was measured by taking 0.1 gm of lyophilized plant sample in 5 mL of reaction buffer containing 10% trichloroacetic acid (TCA) and 0.6% thiobarbituric acid (TBA). The resulting mixture was then centrifuged (12,000 rpm, 20 min). The supernatant was mixed in 2 mL of reaction buffer and kept for incubation at 98° C for 15 min. Again, centrifugation was performed at 12,000 rpm for 10 min. MDA activity was checked (450, 600, and 532 nm) (Li et al., 2010) according to the formula mentioned below:


$$\text{MDA}\;\left(\mu M\;\text{MDA}\;{\text{gm}}^{-1}\;\text{FW}\right)=6.45\times \left(A_{532}-A_{600}\right)\times A_{450}-0.56.$$

### 2.8 Total antioxidant activity

#### 2.8.1 DPPH (1,1- diphenyl-2- picrylhydrazyl)

It was determined by adding 0.21 gm of DPPH to 100 μL methanol followed by incubation at 20° C. This freshly made DPPH sample was then further diluted, and 500 μL of it was mixed in 500 μL samples of varying range of 25–250 μg ml ^−1^ and then further incubated (15 min). OD was checked at 517 nm (Sharma and Bhat, 2009). DPPH activity was evaluated as follows:


%inhibition=(ODA–ODB)/(ODA)×100.

(A: control, B: sample)

### 2.9 Antioxidant enzyme activity

#### 2.9.1 Lipoxygenase (LOX)

Activity of LOX enzyme was determined using the methodology by Axelrod et al. (1981). It is carried out by mixing 50 μL of crude enzyme sample in 2.4 mL of reaction mixture (100 mM potassium phosphate buffer with pH 6.4, and linoleic acid, (10 mM)). OD of solution was measured at 234 nm (Axelrod et al., 1981).

#### 2.9.2 Phenylalanine ammonia lyase (PAL)

It was determined by adopting the methodology by Mori et al. (2001). OD of reaction mixture was measured at 290 nm (37° C, 30 s each). Reaction mixture was made by adding 200 μL of phenylalanine (40 mM) to 200 μL of enzyme extract and 400 μL of 100 mM buffer (Tris–HCL) with pH 8.9 (Mori et al., 2001).

#### 2.9.3 Polyphenol oxidase (PPO)

It was determined by adopting the methodology by Weisany et al. (2012). For doing this, 50 μL of crude enzyme was mixed in reaction solution (0.1 M catechol and, 100 mM potassium phosphate buffer, with pH 5.8). OD of solution was measured at 420 nm (Weisany et al., 2012).

#### 2.9.4 Superoxide dismutase (SOD)

It was determined using the methodology by Dhindsa et al. (1981). For this, 50 μL of crude enzyme was mixed in mixture (0.1 M potassium phosphate buffer with pH 7.9, 3 mM EDTA, 2 mM NBT, 200 mM methionine, and 75 μm riboflavin). The resulting solution was kept at 25 ° C under white light for 15 min, and OD was measured at 560 nm (Dhindsa et al., 1981).

#### 2.9.5 Catalase (CAT)

Estimation of CAT was performed using the procedure adopted by Tyagi et al. (2017). For doing this, shoot sample weighing 100 mg was mixed in 100 mM phosphate buffer (pH 6.5). The resultant mixture was centrifuged at 12,000 rpm (20 min, 4° C). Crude enzyme sample (50 μL) was mixed in H_2_O_2_ phosphate buffer. OD was measured at 240 nm (Tyagi et al., 2017).

### 2.10 Phytohormone production

Phytohormone accumulation in plant was estimated by taking 10 mg lyophilized sample of leaf (FW), and this lyophilized sample was mixed in 1000 μL methanol and 4 μL standard mixture of phytohormones. High-performance liquid chromatography (HPLC, Agilent 1,260) was used to check phytohormone analysis (Agilent Technologies, United States), which was equipped with API tandom mass spectrometer with turbo spray. Phytohormones were separated on Zorbax Eclipse, XDB-C 18 column (4.6 × 50 × 1.8) mm with two mobile phases having 0.05% CH_2_O_2_ in acetonitrile and water. IAA, ET, CK, and SA were analyzed using multiple reaction monitoring. ANALYST 1.6 software was used to analyze bulk data. The linearity of ionization efficiency was validated by analyzing repeated dilution of a standard mixture. Phytohormone level was determined in reference to relevant internal standard (Vadassery et al., 2012).

### 2.11 Atomic absorption spectroscopy (AAS)

As content in shoot and root samples was measured using AAS (Varian AA240 system). Samples were digested using tri acid including sulphuric acid, nitric acid, and perchloric acid (1:5:1) until a translucent mixture was formed. The resulting sample was separated using Whatman filter paper 42 followed by adjusting volume to 18 mL by adding double distilled water. Each test was conducted in five sets, and their findings were denoted as mean ± SD (Bankaji et al., 2023).

### 2.12 Statistical analysis

Every experiment was conducted in five sets. The significant variations were found out by using two-way analysis of variance (ANOVA) and Student’s *t*-test, with *p*-value ≤0.05. Values are mean ± SD of five samples for each group.

## 3 Results

### 3.1 *In vitro* experiment

**Figure 1:**
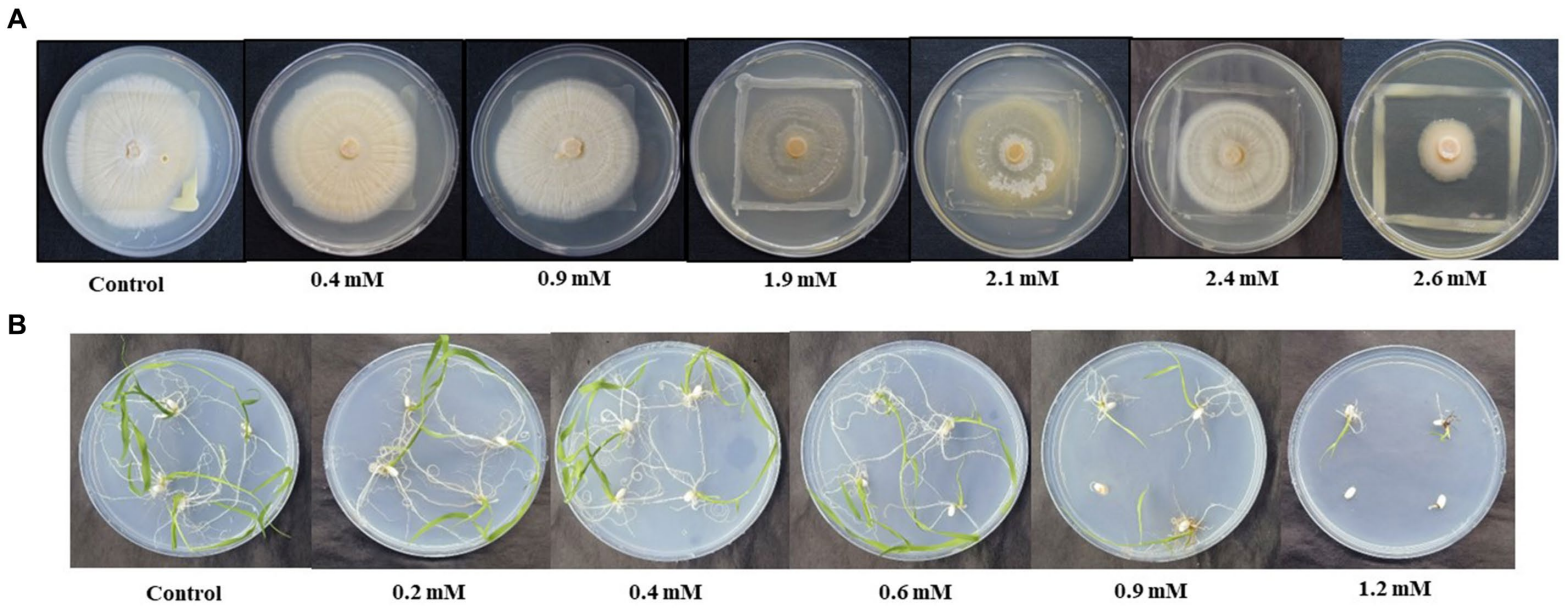
Effect of different concentration of As (V) on **(A)** the growth of combined culture of *Serendipita indica* and *Zhihengliuella* sp. ISTPL4, respectively, under *in vitro* conditions (0.4– 2.6 mM), **(B)** growth of rice seedlings (0.2–1.2 mM).

Growth of combined culture of *S. indica* and *Z.* sp. ISTPL4 was monitored at varying range of As. It was stated that both were able to grow at 2.6 mM concentration of As. Minimum inhibitory concentration (MIC) of both the microbes in combination was 2.6 mM (Figure 1A). Similarly, *in- vitro* growth of germinated rice seedling was also checked at different ranges of As. It was observed that the seedlings were able to germinate at 1.2 mM As concentration (Figure 1B).

### 3.2 Phenotypic traits

**Figure 2:**
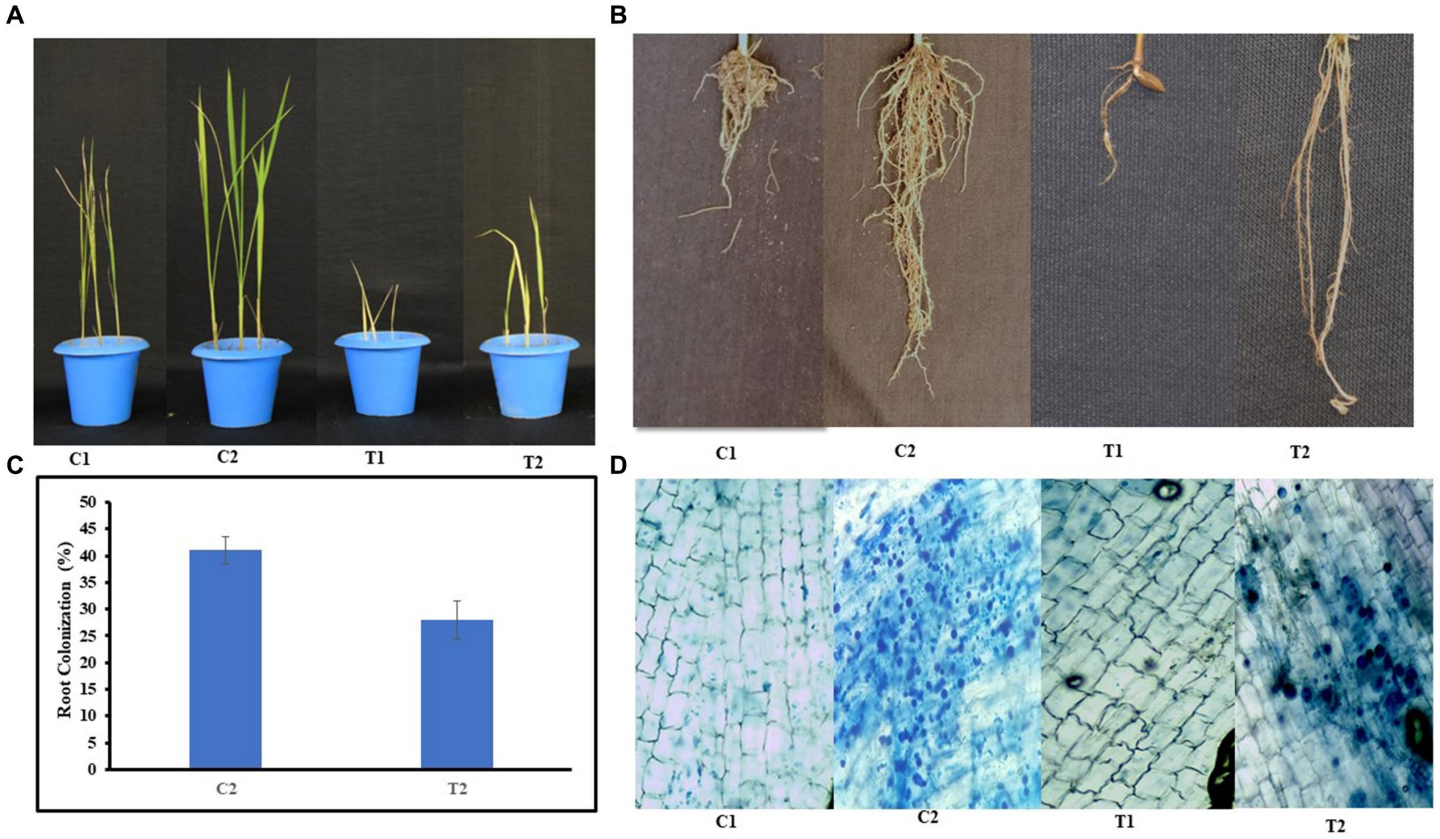
Growth of rice plants **(A)** under normal and microbial-treated conditions (C1: control, C2: inoculated and non-stressed, T1: non-inoculated and stressed, T2: inoculated and stressed), **(B)** root morphology of C1, C2, T1, and T2 plants, **(C)** percent root colonization by *S. indica* spores in C2 and T2 plants, **(D)** microscopic images showing root colonization in C1, C2, T1, and T2 plants, respectively; (values are means of five biological replicates with standard error, *p* < 0.05) (‘*’: *p* ≤ 0.05; ‘**’: *p* ≤ 0.01; and ‘***’: *p* ≤ 0.001).

Plant growth was assessed in pot experiments, and the root morphology of rice plants was also checked (Figure 2A). Root colonization was decreased to 41% in T2 plants when compared with C2 plants (Figures 2B–D).

**Figure 3:**
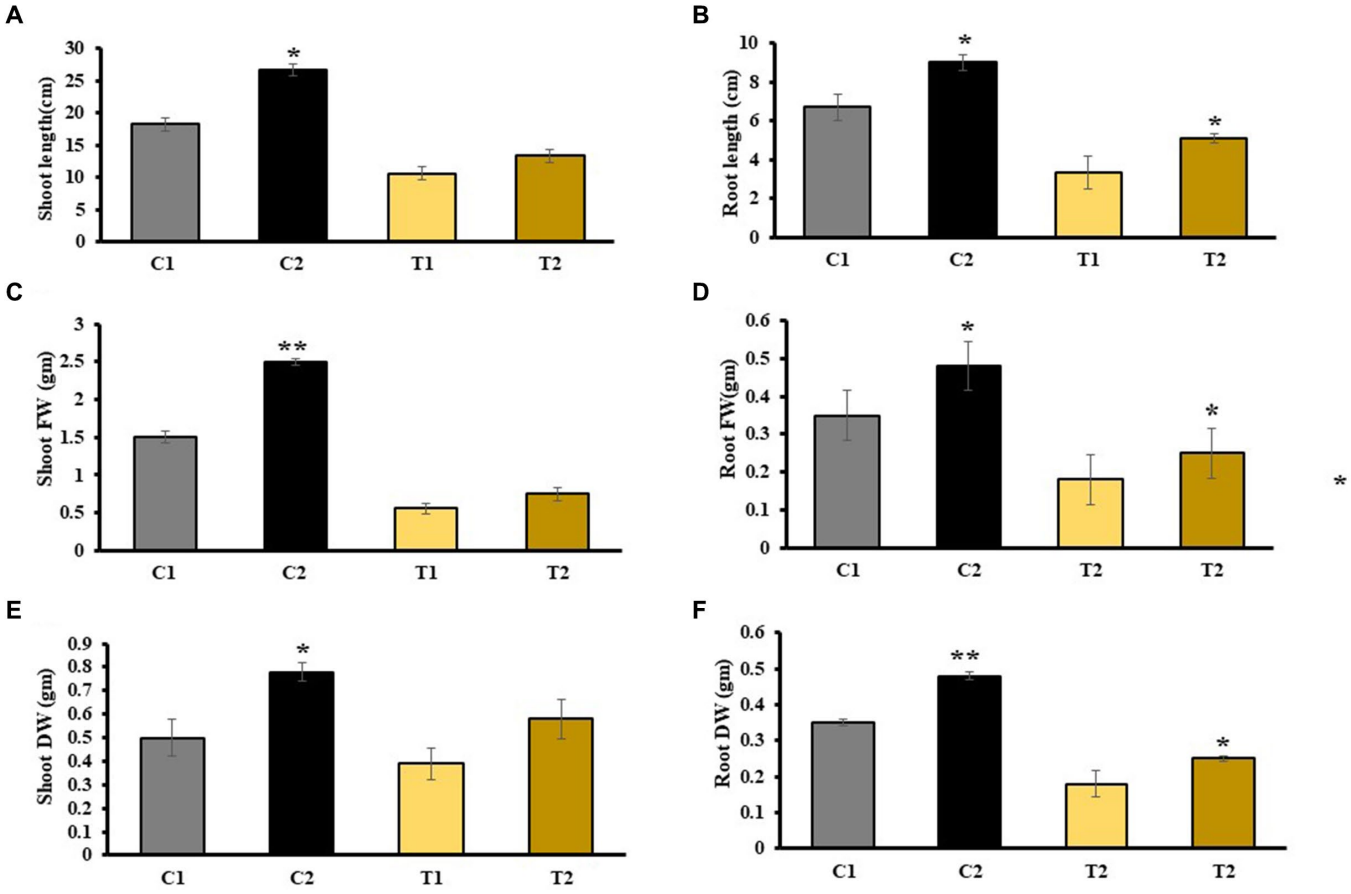
Estimation of various physical parameters including **(A)** SL, **(B)** RL, **(C)** SFW, **(D)** RFW, **(E)** SDW, and **(F)** RDW (values are means of five biological replicates with standard error, *p* < 0.05) (‘*’: *p* ≤ 0.05; ‘**’: *p* ≤ 0.01; and ‘***’: *p* ≤ 0.001).

The results of phenotypic traits indicated that there was 48% increase in SL, and 35% increase in RL of C2 and SL and RL of T2 were increased by 26 and 23% than C1 plants. Similarly, SL of T2 plants was increased by 19% than T1 (Figures 3A,B). SFW and RFW of C2 were assessed, and it was observed that SFW and RFW were 66 and 60% higher than C1 plants, respectively (Figures 3C,D). SDW and RDW of T2 were 33 and 37% higher than that of T1, respectively (Figures 3E,F).

### 3.3 Biochemical traits

**Figure 4:**
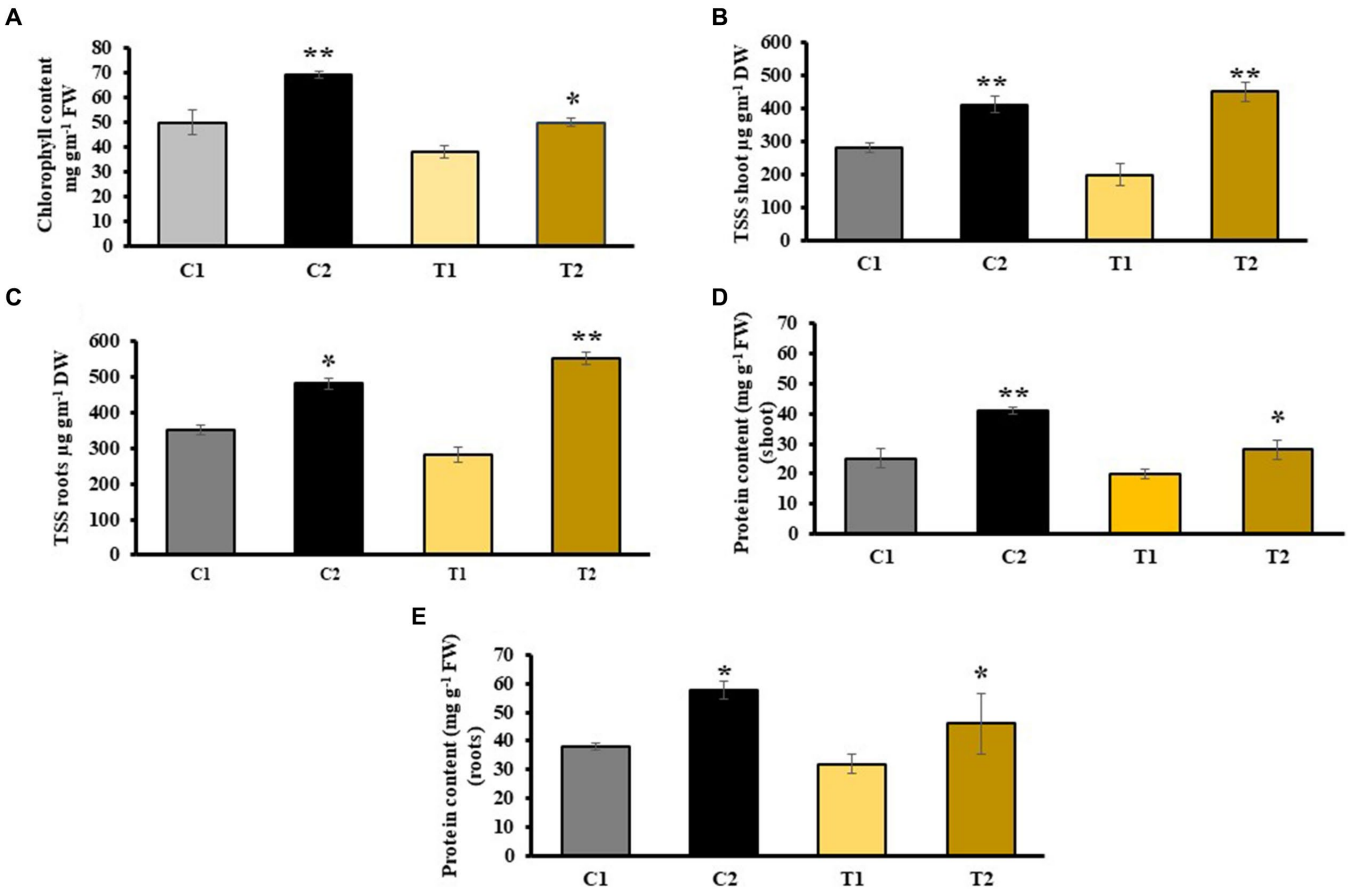
Impact of combined inoculation of *S. indica* and *Z.* sp. ISTPL4 on various biochemical parameters including **(A)** chlorophyll content, **(B)** total soluble sugar (TSS) leaves, **(C)** total soluble sugar (TSS) roots, **(D)** protein content of leaves, **(E)** protein content of roots; (values are means of five biological replicates with standard error, *p* < 0.05) (‘*’: *p* ≤ 0.05; ‘**’: *p* ≤ 0.01; and ‘***’: *p* ≤ 0.001).

The results of chlorophyll content indicated 38% increase in C2 plants than in C1 plants. In T2, chlorophyll content was 22% higher than in T1 (Figure 4A). Similarly, the findings of TSS indicated 109 and 87% more in shoots and roots of C2 than that of C1. TSS of shoots and roots of T2 were 60 and 55% higher than that of T1 (Figures 4B,C), respectively. Total protein content increased by 25.8 and 27% in shoots and roots of C2 than that of C1, whereas it was 37 and 48% higher in shoots and roots of T2 than that of T1, respectively (Figures 4D,E).

### 3.4 Production of secondary metabolites

**Figure 5:**
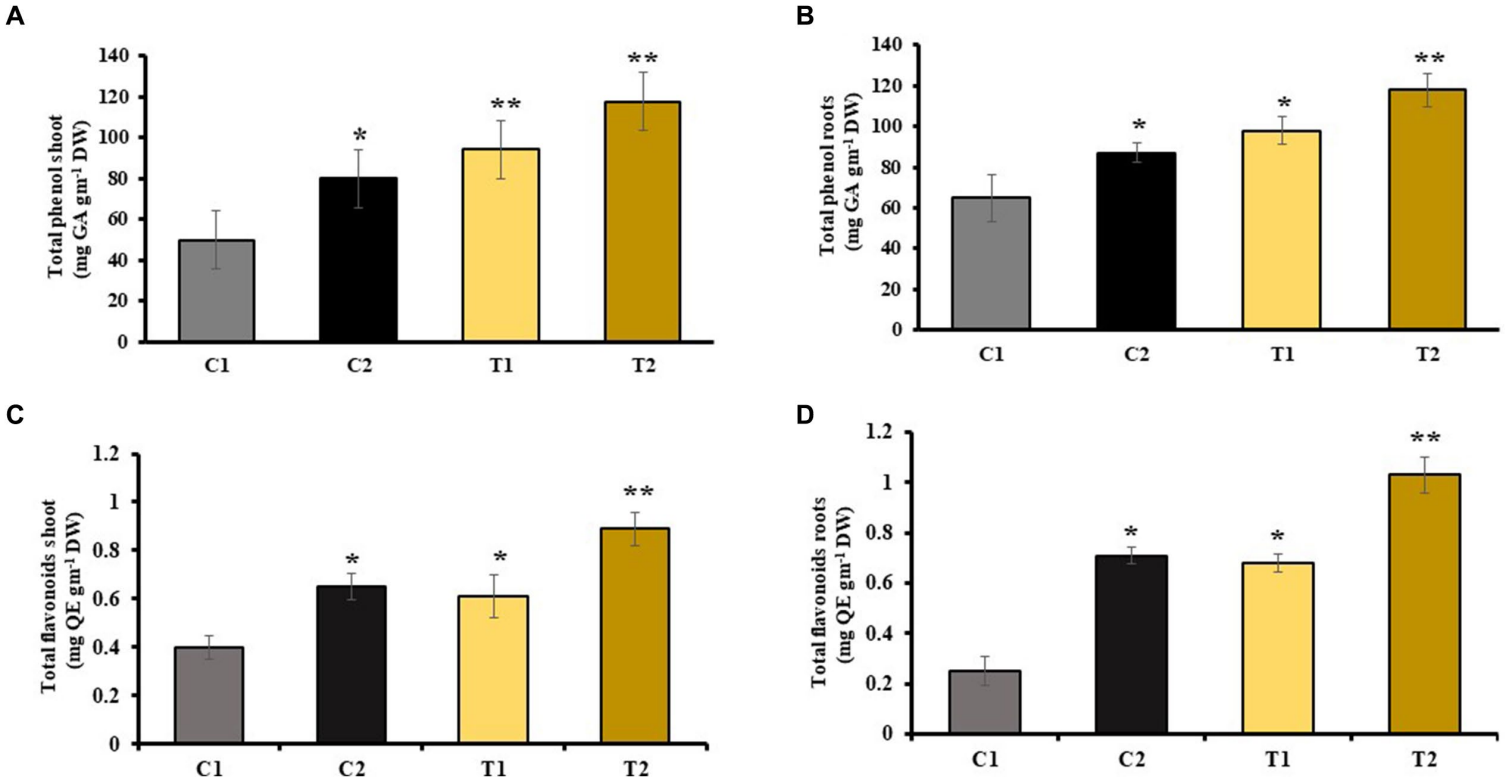
Influence of combined inoculation of *S. indica* and *Z.* sp. ISTPL4 on phenolic and flavonoid contents of **(A)** leaves, **(B)** roots, **(C)** leaves, and **(D)** roots of rice plants; (values are means of five biological replicates with standard error, *p* < 0.05) (‘*’: *p* ≤ 0.05; ‘**’: *p* ≤ 0.01; and ‘***’: *p* ≤ 0.001).

Phenol and flavonoids are secondary metabolites generally released when plants experience stress. They promote plant growth by functioning as antioxidants, protecting against pathogens, and stimulating plant hormone secretion. The analysis of total phenolic content showed that the levels were 22 and 46% higher in the shoot and root, respectively, of C2 compared with C1. Similarly, the analysis of total phenolic content showed that the levels were 34 and 41% higher in the shoots and roots of T2 than that of T1, respectively (Figures 5A,B). The findings of flavonoid content revealed 34 and 54% rise in shoots and roots of C2 than C1, respectively. There was 44 and 69% increase in shoots and roots of T2 than that of C1 (Figures 5C,D).

**Figure 6:**
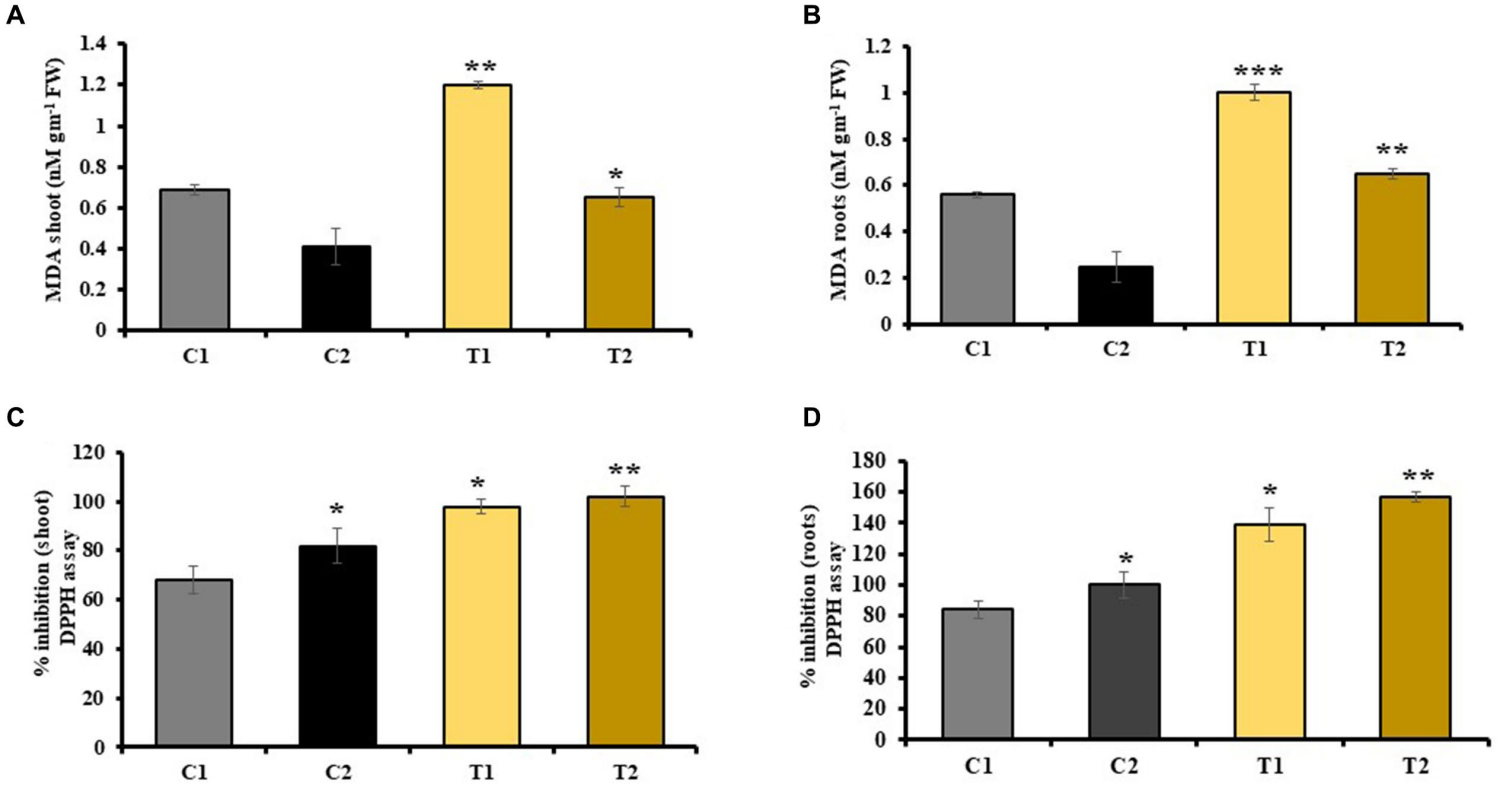
Effect of combined inoculation of *S. indica* and *Z.* sp. ISTPL4 on antioxidant enzyme activities including MDA and DPPH activity in **(A)** leaves, **(B)** roots, **(C)** leaves, and **(D)** roots, respectively; (values are means of five biological replicates with standard error, *p* < 0.05) (‘*’: *p* ≤ 0.05; ‘**’: *p* ≤ 0.01; and ‘***’: *p* ≤ 0.001).

MDA content in shoots and roots of rice plant was checked, which indicated a decrease in MDA by 46 and 60% in shoots and roots of C2 than that of C1. Similarly, it was 52 and 63% less in T2 than T1, respectively (Figures 6A,B).

### 3.5 Antioxidant activities

DPPH activity of plant was also determined, which revealed 40 and 57% increase in DPPH activity of shoots and roots of C2 than that of C1. Similarly, DPPH activity of plant was 22 and 64% higher in shoots and roots of T2 than that of T1, respectively (Figures 6C,D).

### 3.6 Influence of microorganisms on enzymatic antioxidant activity

**Figure 7:**
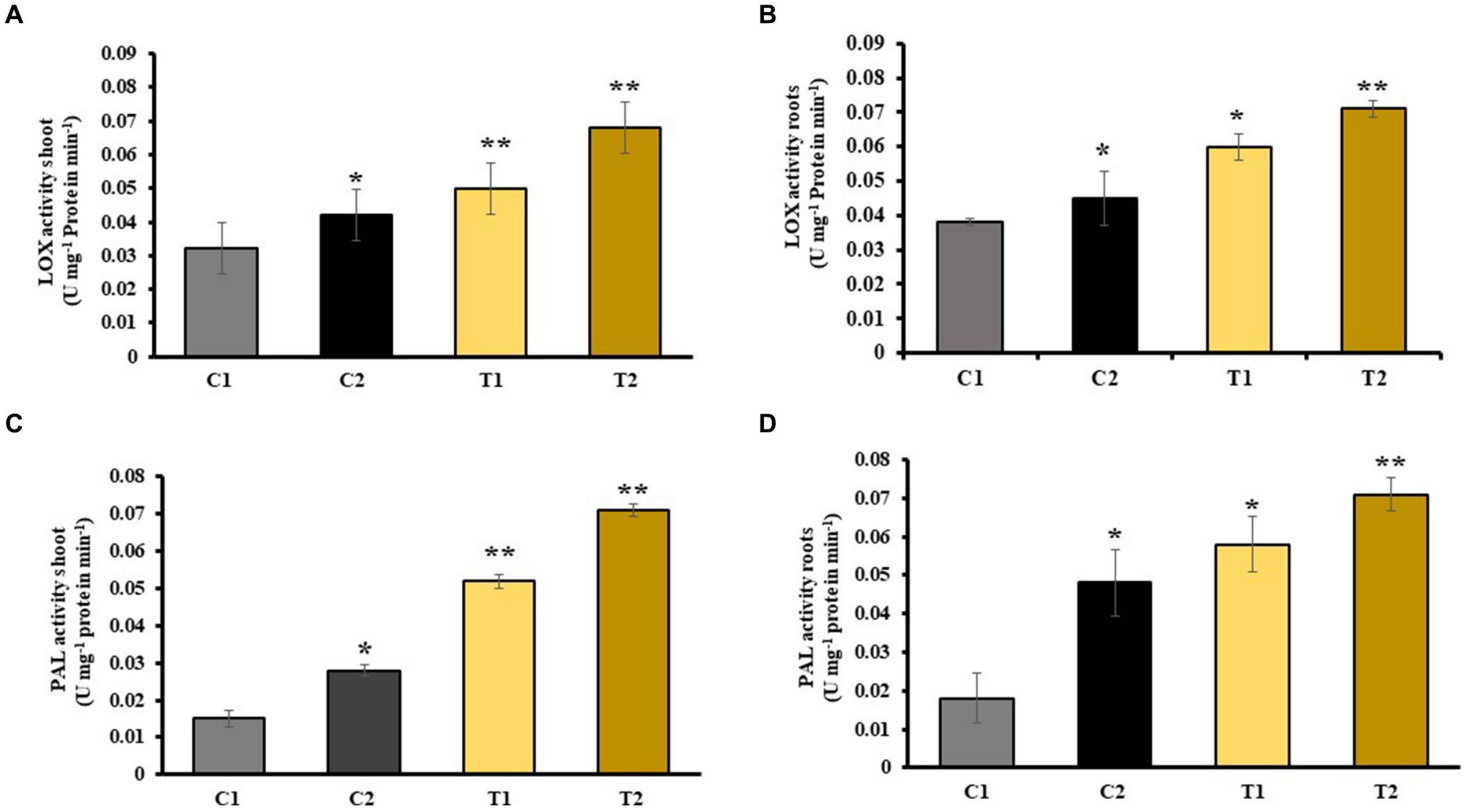
Impact of combined inoculation of *S. indica* and *Z.* sp. ISTPL4 on activities of stress enzyme including LOX and PAL activity of **(A)** leaves, **(B)** roots, **(C)** leaves, and **(D)** roots, respectively; (values are means of five biological replicates with standard error, *p* < 0.05) (‘*’: p ≤ 0.05; ‘**’: *p* ≤ 0.01; and ‘***’: *p* ≤ 0.001).

Activity of LOX enzymes in shoots and roots of C2 was determined which showed 15 and 26% increase than C1. Similarly, it was 57 and 73% high in T2 than in C1. LOX activity of T2 was also observed, and it was 31 and 79% higher than in T1 (Figures 7A,B). An increase in PAL activity of shoots and roots of C2 was reported than C1. It was 62 and 100% higher than C1. Similarly, PAL activity of T2 was 28 and 59% higher than that of C1, respectively, while it was 19 and 34% higher than shoots and roots of T1 (Figures 7C,D).

**Figure 8:**
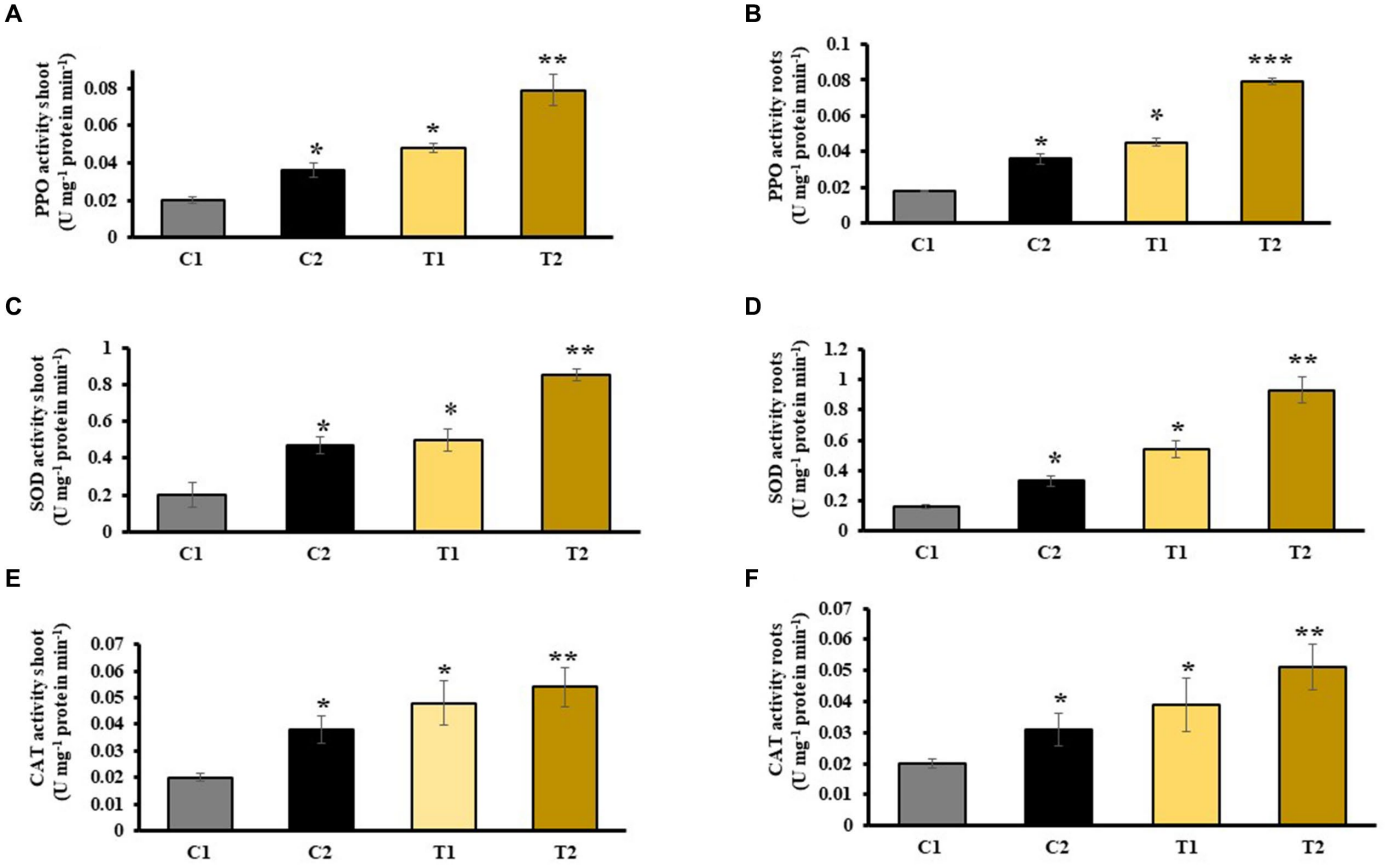
Effect of combined inoculation of *S. indica* and *Z.* sp. ISTPL4 on activities of stress enzyme including PPO, SOD, and CAT activity of **(A)** leaves, **(B)** roots, **(C)** leaves, **(D)** roots, **(E)** leaves, and **(F)** roots, respectively; (values are means of five biological replicates with standard error, p < 0.05) (‘*’: *p* ≤ 0.05; ‘**’: *p* ≤ 0.01; and ‘***’: *p* ≤ 0.001).

An increased PPO activity in shoots and roots of C2 was observed that denoted 44 and 51% rise than C1. Similarly, PPO activity in shoots and root of T2 was 73 and 78% higher than C1, respectively, and 39 and 42% more than T1 (Figures 8A,B). Activity of superoxide dismutase in shoots and roots of C2 was measured, which showed an increase in 51 and 57% than C1, respectively (Figures 8C,D). Catalase activity of shoots and roots of C2 was 43 and 54% more than C1. Similarly, it was 68 and 76% higher in T2 than in C1, respectively. It is 24 and 52% higher in T2 than in T1 (Figures 8E,F).

### 3.7 Phytohormones and heavy metal accumulation

The accumulation of phytohormones in rice under As stress condition was examined, revealing elevated levels of ET, CK, and JA in T2 compared with T1, with concentrations of 4.1, 3.62, and 2.5 μmol mg^−1^ of FW, respectively. Conversely, the concentration of other hormones such as BR was lower in T2 plants than in T1 plants (refer to Supplementary Table S1). Furthermore, melatonin, verbascoside, and ABA were found to be present in higher concentrations in T2 plants, followed by T1 and C2 plants, respectively.

**Figure 9:**
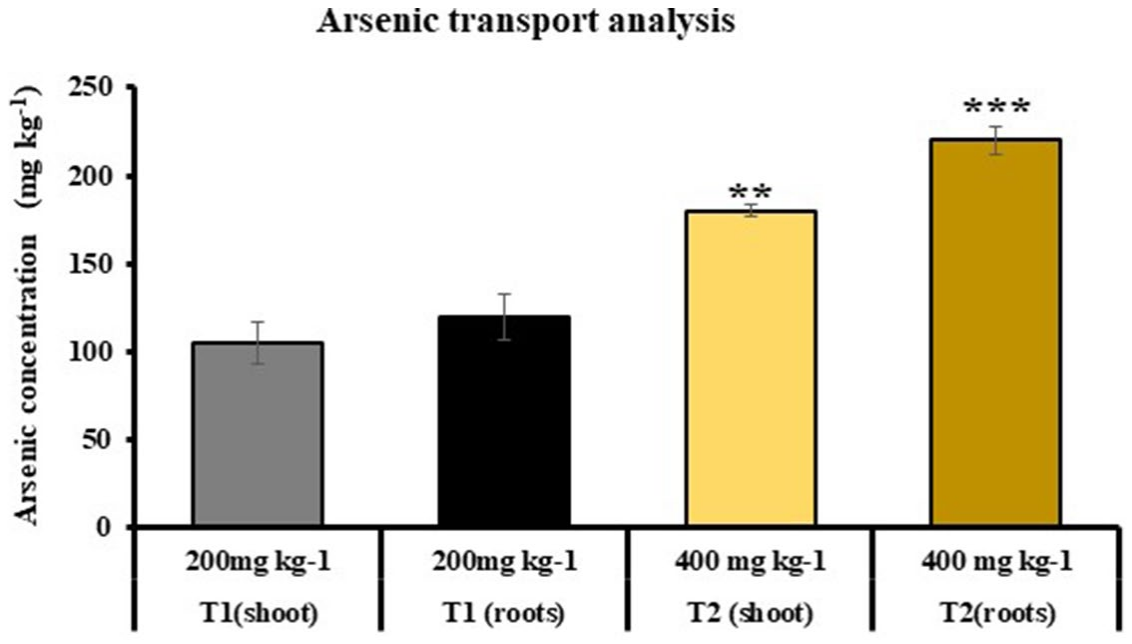
Atomic absorption spectroscopy to determine the As uptake in shoot and roots of T1 and T2 plants, respectively (T1: plants under As stress at 200 mg kg^−1^ concentration in soil and T2: plants grown under As stress and microbial-treated conditions at 400 mg kg^−1^ in soil; growth of T1 plants was not observed at 400 mg kg^−1^ As concentration); (values are means of five biological replicates with standard error, *p* < 0.05) (‘*’: *p* ≤ 0.05; ‘**’: *p* ≤ 0.01; and ‘***’: *p* ≤ 0.001).

Atomic absorption spectroscopy was used to find out As accumulation in T1 and T2 plants grown in soil, which was previously mixed with 200 and 400 mg kg^−1^ of As concentration, respectively. It was observed that As accumulation was 105 and 91 mg kg^−1^ in shoot of T1 and T2 plants and 120 and 131.5 mg kg^−1^ in root of T1 and T2, respectively (at 200 mg kg^−1^ As in soil). Similarly in case of T2, As accumulation was 180 and 220 mg kg^−1^ in shoots and roots, repectively (at 400 mg kg^−1^ As in soil). It was reported that growth of T1 plants was only observed at 200 mg kg^−1^ of As concentration (Figure 9).

## 4 Discussion

To reduce detrimental effects of HMs on agricultural output and manage abiotic stresses, a variety of fungal and bacterial species are helpful (Bilal et al., 2018). In the current investigation, growth of *S. indica* in combination with *Z.* sp. ISTPL4 was studied at varied concentrations of As, and it was discovered that both were able to grow at 2.6 mM range of As (Figure 1A). Similarly, HM tolerance ability was observed in various fungal species, such as *Mucor* and *Trichoderma* (Moya, 2014). The germination of rice seedlings was also determined at varying concentration range of As by carrying out an *in vitro* experiment which indicated better germination at 0.9 mM of As concentration and minimum seedling germination at 1.2 mM of As concentration. Similar experiment was also carried out by researchers to observe the impact of As on rice seedling germination. They reported better growth of rice seedlings at 4 mg As L^−1^, while the germination was minimum at 8 mg As L^−1^ (Abedin and Meharg, 2016).

Rice growth was measured in terms of various physical parameters such as SL, RL, number of shoot, and number of lateral root. As-treated rice plants showed reduction in SL, RL, and biomass than non-stressed plants. Singh et al. (2016) also reported shorter SL and RL of As-treated rice plants when compared with *Brevundimonas diminuta*-treated rice plants (Singh et al., 2016). The results of other study were also consistent with our study, and they reported reduced SFW, SDW, RFW, and RDW in As-stressed plants (Hartley-Whitaker et al., 2001). It may be because roots are primary location for As interaction, and its accumulation in root vacuole resulted in decreased RL. Upadhyay et al. (2016) reported increased SL and RL of rice plants when treated with *Nannochloropsis* sp. and *C. vulgaris* that is similar to our results, which indicated that plants treated with microbial combination (C2) showed significant increase in SL, RL, and number of lateral roots in contrast to control plants (C1).

In the case of T2 plants, an improved SFW and RFW was reported in contrast to T1 plants (31 and 39%, respectively). Similarly, In T2 plants, SDW and RDW were enhanced by 33 and 37%, respectively, than in T1. Root colonization percentage indicated an increased root colonization in C2 than in T2 plants. Similarly, an increase in root colonization percentage was also reported in *Stevia rebaudiana* treated with *P. indica* and *A. chrococcum* than their respective control (Kilam et al., 2015). There was 38% increase in chlorophyll content in C2 compared with C1 and a 22% increase in T2 compared with T1, respectively. Upadhyay et al. (2016) reported higher chlorophyll content in the shoot of rice plants treated with *C. vulgaris* and *Nannochloropsis* sp. in comparison to control plants (Upadhyay et al., 2016). Plants inoculated with *S. indica* exhibit higher chlorophyll levels under As stress possibly due to upregulation in the expression of genes involved in chlorophyll synthesis upon inoculation of *S. indica* (Jogawat et al., 2016). The results of total soluble sugar content were 60 and 55% higher in shoots and roots of T2 than that of T1. It could be due to stress-induced osmotic imbalances, leading to dehydration of various biomolecules and reduction in overall solubility. However, microbes help in increasing total soluble sugar by enhancing the uptake of essential nutrients such as nitrogen, phosphorus, potassium, and carbon. These sugars then serve as antioxidants and osmoprotectants, aiding plants in managing metal stress (Mishra et al., 2016; Khan et al., 2020a,b). In T2 plants, there was 37% increase in protein content in shoot when compared with T1 plants. It might be due to more nutrient availability and low As toxicity.

Phenolic compounds help in protecting plants under stressed conditions (Sharma et al., 2020). They are ROS scavangers that are released during stressed conditions (Corradini et al., 2011). In T2 plants, there was a 34% increase in phenolic content in the shoot and a 41% increase in the root compared with T1 plants. Additionally, the total flavonoid content was 37% higher in shoots and 32% higher in roots of T2 plants compared with T1. Microbes help in stress tolerance in plants by releasing ROS scavengers, such as various antioxidant enzymes, which detoxify ROS that is generated during oxidative stress (Xiao et al., 2020; Wang et al., 2020b). Increased ROS accumulation results in degradation of membrane lipids which resulted in an increased accumulation of MDA as observed in As-treated rice plants (Upadhyay et al., 2016). Our findings indicated a 46 and 60% increase in MDA activity in the shoots and roots of C1 compared with C2 and a 52 and 63% increase in MDA activity in the shoots and roots of T1 compared with T2, respectively.

Various antioxidant enzymes such as LOX, SOD, PAL, PPO, and CAT released in stress conditions and help plant to cope with stress (Ghorbanli et al., 2013). Activity of these stress enzymes was measured in rice plants under As stress. There was 31 and 79% more LOX activity in the shoots and roots of T2 than T1. PAL activity also indicated an increase in T2 than in T1. PPO activity was 39 and 42% higher in shoots and roots of T2 than in T1 plants. It was reported that an increased activity of CAT, PAL, and SOD in rice was observed after the application of SA (Wang F. et al., 2020). Various reports revealed the indirect role of rhizobacteria in increasing the ROS scavenging enzyme activity under As stress (Wang J. et al., 2020; Xiao et al., 2020). In T2 plants, SOD activity in shoots and roots of T2 was increased by 76 and 70% as compared with T1 plants, respectively. Awasthi et al. (2018) noted similar increased SOD activity in rice seedlings that were subjected to treatment with *C. vulgaris* and *Pseudomonas putida* compared with untreated rice plants (Awasthi et al., 2018).

Phytohormones are organic compounds which help in boosting plant growth and increasing plant tolerance to various biotic and abiotic stresses. Stress increases the level of some phytohormones, which function as precursor molecules in signaling pathways (Khan et al., 2020a,b). An increased phytohormone accumulation including IAA, GA, ET, ABA, and CK was reported in T2 plants under As stress. Singh et al. (2019) also reported similar findings, and they observed high accumulation of IAA and CK in microbial-treated *Pisum sativum L*. These hormones activate gene expression involved in secondary metabolite production and take part in molecular signaling pathways (Salvi et al., 2021). In the current study, melatonin concentration increased in T2 plants than T1 and C1 plants, respectively. Accumulation of SA was higher in T2 plants followed by T1 plants. Jan et al. (2019) also reported an increased concentration of SA in microbial-treated Cd-stressed plants as compared with Cd-stressed plants (Jan et al., 2019). It may be because of the secretion of various metabolites and antioxidants by microbes that lower the heavy metal-mediated oxidative stress in plants. Similarly, other phytohormones such as JA and antioxidant-like verbascoside were secreted in higher concentration in T2 plants followed by T1, C2, and C1 plants, respectively. JA and verbascoside are released during ROS-mediated oxidative stress. Following plant exposure to heavy metals, these molecules can increase biomass production and reduce MDA content. They can also lessen oxidative stress caused by ROS by breaking down ROS derivatives such as OH^-^ and H_2_O_2_ in leaves and use oxidative stress to regulate transcriptional pathways (Emamverdian et al., 2015).

In T2 plants, there was more accumulation of As in roots followed by shoot (131.5 and 91 mg kg^−1^), while no major change was observed in As accumulation in shoots and roots of T1 plants (105 and 120 mg kg^−1^, respectively) at 200 mg kg^−1^ concentration of As in soil. It was also observed that T2 plants were able to grow at 400 mg kg^−1^ concentration of As, and no growth of T1 plants was observed. Our results are similar to the findings by Upadhyay et al. (2016), who also reported an increased As accumulation in roots of rice plants treated with microbial combination of *Nannochloropsis* sp. and *C. vulgaris*. The high concentration of As in roots is due to its significant buildup in root vacuoles, where compartmentalization leads to its accumulation.

**Figure 10:**
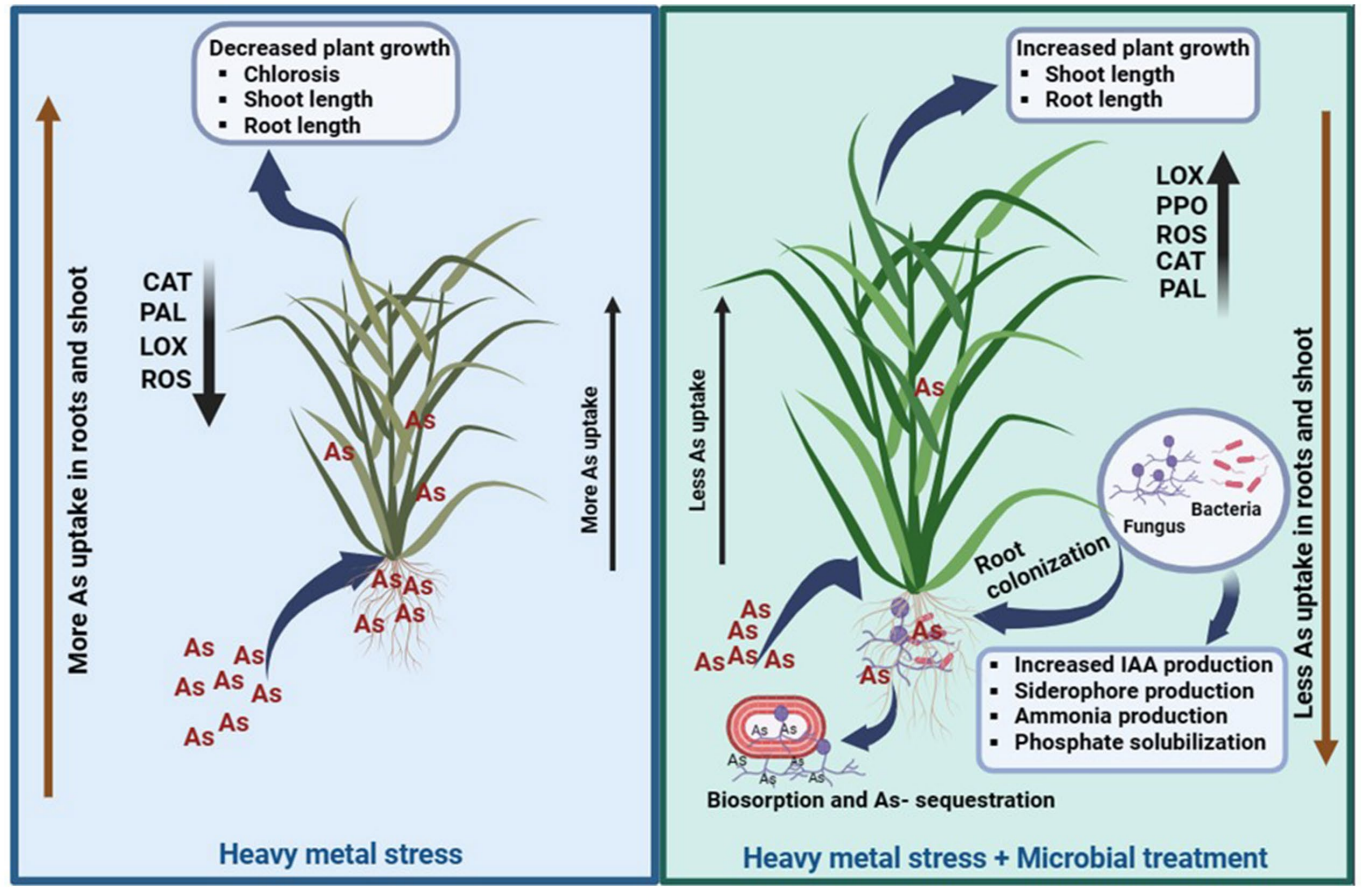
Schematic representation of As uptake in rice plants under control conditions (T1) and in the presence of microbial-treated conditions (T2), Activities of antioxidant enzymes (LOX, PAL, CAL, and SOD) increased in T2 and their activity was comparatively low in T1.

The uptake of As in rice plants under control (T1) and microbial-treated plants (T2) was described according to the mechanism explained (Figure 10). It was observed that in T1 plants, As uptake is more in shoots followed by roots. The uptake of As in shoot is comparatively lower than As accumulation in roots of T2 plants. This could be attributed to the capacity of this particular combination of microbes to absorb As. Earlier As uptake ability of *S. indica* was reported, and it was observed that this root endophytic fungus has certain genes which are involved in As bioaccumulation and biotransformation (Mohd et al., 2017; Sharma et al., 2024). Similarly, HM tolerance ability of this actinobacterium *Zhihengliuella* sp. ISTPL4 was reported by Mishra et al. (2020). The results of whole genome sequencing of *Zhihengliuella* sp. ISTPL4 also revealed the presence of arsenate reductase gene *arsC* (Mishra et al., 2018). It can be inferred from this study that this microbial combination holds promise for enhancing agricultural output and could serve to counteract the harmful impacts of As in polluted areas.

## 5 Conclusion

This study concludes that the overall health of *Oryza sativa* can be improved by combined inoculation of *S. indica* and *Z.* sp. ISTPL4. Our research findings also indicate that the oxidative stress caused by As can be alleviated by employing this microbial combination. Plants treated with this microbial combination in the presence of As stress exhibited heightened levels of phytohormones and increased activity of antioxidant enzymes. These enzymes are crucial in mitigating the oxidative stress induced by As. Similarly, phytohormones such as ABA, CK, ET, SA, and BR play important roles in promoting plant growth and protecting plants against various environmental stresses. Utilizing this microbial combination could prove beneficial in enhancing sustainable agricultural practices, particularly in fields contaminated with As.

## Data availability statement

The datasets presented in this study can be found in online repositories. The names of the repository/repositories and accession number(s) can be found in the article/Supplementary material.

## Author contributions

NS: Formal analysis, Investigation, Methodology, Writing – original draft. GY: Formal analysis, Methodology, Visualization, Writing – review & editing. JT: Formal analysis, Visualization, Writing – review & editing. AK: Data curation, Formal analysis, Visualization, Writing – review & editing. MK: Formal analysis, Supervision, Writing – review & editing. NJ: Conceptualization, Project administration, Supervision, Writing – review & editing. AH: Formal analysis, Funding acquisition, Visualization, Writing – review & editing. EA: Formal analysis, Visualization, Writing – review & editing. AM: Conceptualization, Methodology, Supervision, Visualization, Writing – review & editing.
