# Supplementary material for: Synergistic impact of Serendipita indica and Zhihengliuella sp. ISTPL4 on the mitigation of arsenic stress in rice
Source: Front Microbiol. 2024 May 29;15:1374303. doi: 10.3389/fmicb.2024.1374303 (PMC11168111; doi:10.3389/fmicb.2024.1374303)
Supplement: Supplementary file 1 [file Table_1.docx]

***Supplementary Material***

**Synergistic impact of *Serendipita indica* and *Zhihengliuella* sp. ISTPL4 on the mitigation of arsenic stress in rice**

Neha Sharma^1^, Gaurav Yadav^1^, Jaagriti Tyagi^1^, Ajay Kumar^2^, Monika Koul^3^, Naveen Chandra Joshi^1^*, Abeer Hashem^4^, Elsayed Fathi Abd_Allah^5^, Arti Mishra^3^*

1. Amity Institute of Microbial Technology, Amity University, Noida, Uttar Pradesh, India,

2. Amity Institute of Biotechnology, Amity University, Noida, Uttar Pradesh, India,

3. Department of Botany, Hansraj College, University of Delhi, New Delhi, India,

4. Botany and Microbiology Department, College of Science, King Saud University, Riyadh, Saudi Arabia,

5. Plant Production Department, College of Food and Agricultural Sciences, King Saud University, Riyadh, Saudi Arabia

**Corresponding authors:** Naveen Chandra Joshi and Arti Mishra

**Table S1.** Phytohormones accumulation in rice inoculated with a combination of *S. indica* and *Z.* sp. ISTPL4 under normal conditions as well as in As stressed condition

| **S. No** | **Phytohormones**  **(µmol mg^-1^ of FW)** | **Concentration**  **(C1)** | **Concentration**  **(C2)** | **Concentration**  **(T1)** | **Concentration**  **(T2)** |
| --- | --- | --- | --- | --- | --- |
| **1.** | **Auxin** | 1.1 ± 0.009 | 1.5± 0.005 | 0.4± 0.03 | 0.8± 0.04 |
| **2.** | **Gibberellins** | 0.4± 0.0004 | 0.6± 0.003 | 1.9± 0.01 | 2.5 ± 0.04 |
| **3.** | **Cytokinin** | 1.2± 0.0009 | 1.7±0.02 | 1.5± 0.009 | 3.6± 0.01 |
| **4.** | **Ethylene** | 0.003± 0.001 | 3.6± 0.18 | 1.2±0.018 | 4.1± 0.102 |
| **5.** | **Abscisic acid** | 1.5± 0.01 | 2.9± 0.01 | 2.5± 0.07 | 4.6± 0.1 |
| **6.** | **Salicylic acid** | 0.5± 0.01 | 0.2± 0.08 | 0.35±0.002 | 1.05± 0.001 |
| **7.** | **Jasmonic acid** | 0.01± 0.0002 | 0.06± 0.0003 | 0.09± 0.002 | 2.5± 0.03 |
| **8.** | **Brassinosteroids** | 0.0003±0.0001 | 0.0008± 0.0004 | 0.02± 0.002 | 0.002± 0.005 |
| **9.** | **Strigolactones** | 0.0002±0.0001 | 0.0005±0.0002 | 0.001± 0.0002 | 0.001 ±0.0004 |
| **10.** | **Melatonin** | 0.004±0.0002 | 0.2±0.002 | 0.7± 0.06 | 2.5 ± 0.06 |
| **11.** | **Verbascoside** | 0.0004±0.0002 | 0.003±0.0004 | 1.3± 0.1 | 3.5 ± 0.08 |
